# Comparing changes in haematologic parameters occurring in patients included in randomized controlled trials of artesunate-amodiaquine *vs *single and combination treatments of uncomplicated falciparum in sub-Saharan Africa

**DOI:** 10.1186/1475-2875-11-25

**Published:** 2012-01-25

**Authors:** Julien Zwang, Jean-Louis Ndiaye, Abdoulaye Djimdé, Grant Dorsey, Andreas Mårtensson, Corine Karema, Piero Olliaro

**Affiliations:** 1 Drugs for Neglected Diseases Initiative (DNDi), Geneva, Switzerland; 2 Department of Parasitology, Faculty of Medicine, Cheikh Anta Diop University, Dakar, Senegal; 3 Malaria Research and Training Center, Department of Epidemiology of Parasitic Diseases, Faculty of Medicine and Pharmacy, University of Bamako, Bamako, Mali; 4 Department of Medicine, University of California San Francisco, San Francisco, CA, USA; 5 Infectious Diseases Unit, Department of Medicine, Karolinska University Hospital, Karolinska Institutet, Stockholm, Sweden; 6 Division of Global Health (IHCAR), Department of Public Health Sciences, Karolinska Institutet, Stockholm, Sweden; 7 Centre National de Recherche et de Formation sur le Paludisme, Ministère de la Santé, Ouagadougou, Burkina Faso; 8 UNICEF/UNDP/WB/WHO Special Programme for Research & Training in Tropical Diseases (TDR), Geneva, Switzerland; 9 Centre for Tropical Medicine and Vaccinology, Nuffield Department of Medicine, University of Oxford, Churchill Hospital, Oxford OX37LJ, UK

**Keywords:** *Plasmodium falciparum*, Haematology, Artesunate, Amodiaquine, Randomized controlled trial, Sub-Saharan Africa

## Abstract

**Background:**

Artesunate-amodiaquine (AS&AQ) is a widely used artemisinin combination therapy (ACT) for falciparum malaria. A comprehensive appreciation of its effects on haematology *vs *other anti-malarials is needed in view of potential safety liabilities.

**Methods:**

Individual-patient data analysis conducted on a database from seven randomized controlled trials conducted in sub-Saharan African comparing AS&AQ to reference treatments in uncomplicated falciparum malaria patients of all ages. Haematologic values (white cells total and neutrophil counts, haemoglobin/haematocrit, platelets) were analysed as both continuous and categorical variables for their occurrence, (severity grade 1-4) and changes during follow-up. Risks and trends were calculated using multivariate logistic random effect models.

**Results:**

4,502 patients (72% < 5 years old), from 13 sites in nine countries with 28-day follow-up were treated with AS&AQ (45%) or a comparator (other forms of ACT accounted for 27%, other combination 12%, mono-therapies 16%). Pre-treatment leucopaenia (3%) and neutropaenia (6%) were infrequent; anaemia was common (39%). The treatment-emergent adverse events incidence (TEAE = condition not present or less severe pre-treatment) was 11% for neutropaenia, 6% for thrombocytopaenia with AS&AQ and not different from treatment groups; anaemia was higher with AS&AQ (20%) or other forms of ACT (22%) than in non-artemisinin groups (4%, *p *= 0.001). Multivariate analysis showed that the risk of anaemia, thrombocytopaenia, and leucopaenia decreased with follow-up time, while neutropaenia increased; the risk of anaemia and thrombocytopaenia increased with higher baseline parasitaemia and parasitological reappearance. White cells total count was not a good surrogate for neutropaenia. No systematic significant difference between treatments was detected. Older patients were at lower risks.

**Conclusion:**

The effects of AS&AQ on haematologic parameters were not different from those of other anti-malarial treatments used in sub-Saharan Africa. This analysis provides the basis for a broader evaluation of haematology following anti-malarial treatment. Continuing monitoring of haematologic safety on larger databases is required.

## Background

Artemisinin-based combinations (ACT)-the treatment of choice for uncomplicated *Plasmodium falciparum *malaria [1]-are generally safe and well tolerated, but haematologic toxicity remains of potential concern, in particular for treatments containing amodiaquine. Neutropaenia and agranulocytopaenia have been reported in the past with intermittent (weekly) doses of amodiaquine for malaria prophylaxis; the reported rate of serious events (blood dyscrasias) in the UK was 1:2,100 users, with a fatality rate of 1:75,000 [2]. It has been shown that agranulocytosis is unlikely to occur when amodiaquine is used for treatment (as opposed to prophylaxis), but it is not easy to derive information from published information. A meta-analysis of comparative and non-comparative trials of amodiaquine for treating malaria did not show a particular risk of neutropaenia associated with amodiaquine [3]; other systematic reviews have little safety data, especially on haematologic toxicity [4]. Neutropaenia has been reported after administration of treatment doses of amodiaquine, alone or in combination with artesunate [5-8], and also with artesunate with a dose-dependent risk [9].

Artesunate combined with amodiaquine (AS&AQ) is the second most widely used ACT, adopted as first-line treatment in 18 countries. Over time, AS&AQ has been available in a non-fixed formulation (AS+AQ, as either a loose combination or in blister packs produced by different manufacturers), and more recently, as a fixed-dose, WHO-prequalified, co-formulation (ASAQ).

Although serious adverse events following ACT seem to be uncommon, very few trials compared the haematologic variations between treatments. Two randomized controlled trials (RCT) conducted in sub-Saharan Africa reported no difference in neutropaenia between artesunate-amodiaquine and artemether-lumefantrine [10,11].

Safety in general, and laboratory data in particular, are underreported in malaria trials; risk should be assessed comparatively; databases should be large enough and representative of the spectrum composition of patients and conditions. Limited information can be derived from aggregated data, and individual patient data are best suited for such assessment. The widespread use of these anti-malarial combinations calls for a comprehensive synthesis of available individual patient's haematologic data.

## Methods

Data on age, parasitaemia, haematologic parameters (white blood cells total counts (WCC), neutrophil and lymphocyte counts, haemoglobin or haematocrit and platelet counts), treatment and treatment outcome were extracted from a database of randomized controlled trials (RCT) including AS&AQ groups conducted in sub-Saharan Africa with 28-day follow-up (26 trials in 16 countries and 33 sites with 11,700 participants). Data were censored when patients dropped out or had recurrent *P. falciparum*. Details on these studies are provided elsewhere [12].

The criteria for selection of the seven RCT were based on the presence of haematologic data (n = 4,502) and comparators including monotherapies: amodiaquine (AQ) or artesunate (AS) alone or other artemisinin combination therapy: artemether-lumefantrine (AL), artesunate plus sulphadoxine/pyrimethamine (AS+SP), dihydroartemisinin-piperaquine (DP) or non-artemisinin containing combinations (AQ+SP) [5,13-17](Table 1).

**Table 1:** Number of patients recruited by treatment group and study

RCT	Location	Reference	ACT				Mono-therapy		non-ACT	Total
			AS&AQ	AL	DP	AS+SP	AQ	AS	AQ+SP	
ACT vs monotherapy	Multicentre	[5]	306				307			613
	Rwanda	[17]	158				150			308
	
	Mali	[13]	252			249		252		753
ACT vs ACT and non-ACT	Uganda	[14]	242	217					269	728
	Rwanda	[15]	252		251				258	761
	Multicentre	[16]	626	311						937
	Zanzibar	[10]	202	200						402

Total			2038	728	251	249	457	252	527	4502

Analysis involved 4,502 patients: 10,677 records in 3,829 patients at various time points for WCC; 8,232 records in 3,069 patients for neutrophils; 12,888 records in 4,276 patients for haemoglobin; and 3,514 records in 1,419 patients for platelets.

### Treatments

#### AS&AQ treatment regimens

AS&AQ products were either loose (AS+AQ) or fixed-dose co-formulations (ASAQ). The loose AS+AQ was dosed based on body weight, while the co-formulated ASAQ was based on age and weight range.

The proportion of patients treated with AS&AQ was 45% (2,038/4,502) of which 31% (624/2,038) were treated with a fixed-dose ASAQ combination (Coarsucam™, Winthrop), and 69% (1,412/2,038) were treated with loose combinations: in Gabon, Kenya and Uganda was AS (Arsumax™ 50 mg, Sanofi) and AQ (Camoquine™ 200 mg, Parke-Davis); in Zanzibar: AS (Plasmotrim™ 100 mg, Mepha) and AQ (Flavoquin™, 153 mg, Roussel); in Rwanda (Arsumax™, Sanofi [15]), and AQ and AS (Dafra Pharma [17]).

The loose combination target dose was AS 12 mg/kg over three days and AQ 30 mg/kg over 3 days, except in Uganda where AQ was given at 25 mg/kg (10 mg/kg on Days 0 and 1 then 5 mg/kg on Day 2). The fixed-dose combinations of ASAQ (Coarsucam™, Winthrop) were available as two-and three-strength products given by age. The fixed-dose combination was given either once or twice a day. For the two-strength fixed-dose combination ASAQ (paediatric AS 25 mg+AQ 67.5; adult AS 100 mg+AQ 270 mg, dose ratio = 2.7), the dosing categories were: (i) 0-1 months: 1/2 paediatric; (ii) 2-11 months: 1 paediatric; (iii) 1-6 years: 2 paediatric; (iv) 7-13 years: 1 adult; and (v) ≥ 14 years: 2 adults. For the three-strength ASAQ, age- and weight-based doses were administered once-a-day for three days: one tablet/day for children up to 13 years of age (≤ 35 kg) or two tablets/day for adolescents aged 14 years and above and adults (≥ 36 kg). Doses available were: infants (two to 11 months) received AQ 25 mg/AS 67.5 mg; young children (one to 4 years) received AQ 50 mg/AS 135 mg; children (six to 13 years) received one tablet/day of AQ 100 mg/AS 270 mg, and adults (14 years or more) received two tablets (AQ 100 mg/AS 270 mg) per day.

#### Other forms of ACT

Patients treated with other forms of ACT accounted for 27% (1,228/4,502) of the total: 728 with AL (20 mg artemether/120 mg lumefantrine given according to weight as one (5-14 kg), two (15-24 kg), three (25-34 kg), and four 4 (≥ 35 kg) tablets given twice daily for three days); 251 with DP (around 2.3 mg/kg/day dihydroartemisinin and 18.4 mg/kg for three days); and 249 with AS+SP (AS 4 mg/kg/day; SP 25 mg/kg of sulphadoxine and 1.25 mg/kg/of pyrimethamine administered in a co-formulated tablet (SP) as a single dose).

#### Non-ACT combinations

Patients treated with a non-artemisinin containing combination accounted for 12% (527/4,502): AQ+SP (AQ 10 mg/kg/day for three days and SP 25 mg/kg of sulphadoxine and 1.25 mg/kg/of pyrimethamine administered in a co-formulated tablet (SP) as a single dose).

#### Monotherapies

AQ only (10% of patients) was given at 10 mg/kg/day for three days; AS only (6% of patients) was given at a total dose of 12 mg/kg over five days.

### Haematology

The grading of all paediatric haematological values was derived from international standards [18-20].

Leucopaenia was defined as white blood cell counts (WCC) < 3 × 10^9^/L; mild/moderate was 3 × 10^9^/L to 2 × 10^9^/L, and severe/very severe leucopaenia was < 2 × 10^9^/L (grade 3: 1.9 × 10^9^/L to 1 × 10^9^/L and 4: < 1 × 10^9^/L).

Neutropaenia was defined as neutrophil counts < 1.20 × 10^9^/L; mild/moderate neutropaenia ranged from 1.19-0.40 × 10^9^/L (grade 1: 1.19 × 10^9^/L to 0.75 × 10^9^/L and 2: 0.74 × 10^9^/L to 0.40 × 10^9^/L), and severe/very severe neutropaenia was < 0.40 × 10^9^/L (grade 3: 0.39 × 10^9^/L to 0.25 × 10^9^/L and 4: < 0.25 × 10^9^/L).

For anaemia the cut off was set at haematocrit < 30% or haemoglobin < 10 g/dL and was categorized as: mild/moderate from 9.9-8.0 g/dL of haemoglobin (grade 1: 9.9 g/dl to 9.0 g/dL and 2: 8.9 g/dL to 8 g/dL), and severe/very severe < 8 g/dL of haemoglobin (grade 3: 8 g/dL to 5 g/dL and 4: < 5 g/dL).

Thrombocytopaenia was defined as platelets count < 150 × 10^9^/L; mild/moderate ranged 150-50 × 10^9^/L (grade 1: 149 × 10^9^/L to 75 × 10^9^/L and 2: 74 × 10^9^/L to 50 × 10^9^/L), and severe/very severe thrombocytopaenia < 50 × 10^9^/L (grade 3: 49 × 10^9^/L to 20 × 10^9^/L and 4: < 25 × 10^9^/L).

Recovery from an abnormal condition was defined for leucopaenia as WCC becoming ≥3 × 10^9^/L, for neutropaenia as ≥1.2 × 10^9^/L, for anaemia as haematocrit becoming ≥30% or haemoglobin ≥10 g/dL, for thrombocytopaenia as platelets ≥150 × 10^9^/L.

A haematological adverse event (AE) was defined as the occurrence of an abnormal value (grade 1 or more, as defined above) after treatment start independent of the pre-treatment value. In this analysis, all visits were considered, thus a subject could have multiple AEs.

A treatment-emergent AE (TEAE) expresses the worsening of the condition-i.e. any occurrence of an abnormal value at any follow-up visit (days 7 through 28) as compared to baseline in patients who either had a normal condition pre-treatment or an abnormal value that was of lower grade than post-treatment. Drug-event relationship could not be attributed in the present analysis.

### Statistical analysis

Haematologic changes were analysed between Days 0-7, 0-14, and 0-28 using t-student paired analysis and presented as relative difference (Day 0 as the reference). In each RCT, the patients' paired differences were compared between treatment groups using the Mann-Whitney rank test. Categorical data were compared using a chi-square test or a Fischer exact test or a Mantel-Haenszel chi-square test stratified by site and the comparison presented by odds ratio (OR), as appropriate.

During the 28-day follow-up, the timing of post-treatment haematologic assessments varied across studies (Table 2), therefore an adjustment for time (day of observation) was included in the multivariate analysis. All available haematologic values for every patient were considered. A random intercept for each individual was included when the Lagrange multiplier (LM) test [21] was significant for heterogeneity in multivariate logistic regression. These multivariate analyses assessed the risks for leucopaenia, neutropaenia, anaemia, and thrombocytopaenia (analysed as binary variables) during follow-up. The adjusted risks (AOR) of the above conditions were assessed over time (continuous, in days), according to the patients' age (continuous, in years), parasitaemia (continuous, in parasites/μL, log-transformed), parasitological reappearance (or treatment outcome, including recrudescence and re-infections; binary, at the exact day of occurrence) along with the interaction of age and parasitaemia, as well as the potential effects of the various treatments in each RCT.

**Table 2:** Follow-up timing per study and haematologic parameters

Location	Reference	N	White blood cell counts (WCC)	Neutrophils	Haemoglobin	Platelets
Multi-centre	[5]	613	D0		D0, D28	
Multi-centre	[16]	937	D0, D7, D28	D0, D7, D28	D0, D28	
Mali	[13]	753	D0, D7, D14, D28		D0, D7, D14, D28	D0, D7, D14
Rwanda	[17]	308			D0, D14, D28	
Rwanda	[15]	761	D0, D14	D0, D14	D0, D14	
Uganda	[14]	728	D0, D14	D0, D14	D0, D14	D0, D14
Zanzibar	[10]	402	D0, D7, D14, D21, D28	D0, D7, D14, D21, D28	D0, D7, D14, D21, D28	

Legend: *D *day

For relative differences, confidence intervals (CI) were calculated using the normal distribution. All CIs were calculated at 95% (95%CI) and comparisons considered significant when *p *< 0.05. Data were analysed using Stata v10 (Stata Corp.).

### Ethical issues

All the studies had been approved by the relevant ethics and institution review committees [5,10,13-17].

## Results

The main baseline characteristics of the patients enrolled are displayed in Table 3. Seventy-two percent (72%, range 32-100%) were children under five years of age from 13 sites in nine countries. The geometric mean pre-treatment parasitaemia was 18,425 overall and ranged from 9,203 to 30,988 parasites/μL, by site. At presentation, leucopaenia and neutropaenia were infrequent (3% and 6% respectively), while thrombocytopaenia and anaemia were found in 32% and 49% of subjects (Table 4).

**Table 3:** Age and parasitaemia on admission by site

	N	Parasitaemia (μ/L)	Age (year)			
**Site**	**total**	**geometric mean**	**Under five years old**	**median**	**minimum**	**maximum**

Cameroon	166	24627	44%	6.2	1.0	65.0
Gabon	216	22597	42%	5.4	1.3	10.8
Kenya	397	30988	80%	2.0	.5	11.0
Madagascar	178	9203	32%	7.5	1.4	53.1
Mali-Bancouna	201	22386	54%	4.7	.9	24.3
Mali-Bougoula	753	15468	91%	2.5	.6	13.7
Rwanda-kicukiro	313	27938	99%	3.0	.6	5.0
Rwanda-mashesha	391	18462	99%	3.0	.5	5.0
Rwanda-rukara	365	33615	100%	2.0	.5	4.9
Senegal	392	19142	50%	5.0	.9	64.0
Uganda-Kampala	728	10552	37%	6.1	1.1	11.5
Zanzibar-Kivunge	297	16500	97%	2.5	.5	6.5
Zanzibar-Micheweni	105	16777	99%	2.1	.5	5.0

Total	4502	18425	72%	3.4	.5	65.0

**Table 4:** Haematology on admission by site

	WCC(*10^9^/L)					Neutrophils(*10^9^/L)					Haemoglobin (g/dL)					Platelets(*10^9^/L)				
**Site**	**N**	**mean**	**SD**	**n**	**Leuco-poenia**	**N**	**mean**	**SD**	**n**	**Neutro-paenia**	**N**	**Mean**	**SD**	**n**	**Anae-mia**	**N**	**mean**	**SD**	**n**	**Throm-bocyto-paenia**

Cameroon	166	7.5	1.7	0	0%	165	4.0	1.4	3	2%	166	12.1	1.4	153	8%					
Gabon	216	7.8	3.0	2	1%	207	3.7	2.4	16	8%										
Kenya	41	9.6	3.7	0	0%	41	6.2	3.0	0	0%	397	9.3	2.0	155	61%					
Madagascar	178	7.1	3.0	0	0%	167	4.6	2.7	1	1%	178	11.5	2.3	145	19%					
Mali-Bancouna	201	9.4	3.7	1	0%	194	6.2	3.1	1	1%	200	9.9	1.6	99	51%					
Mali-Bougoula	752	10.4	4.4	4	1%						752	10.2	1.8	424	44%	697	180	93	264	38%
Rwanda-kicukiro	222	6.7	2.6	5	2%	222	3.7	2.0	10	5%	312	10.8	2.0	222	29%					
Rwanda-mashesha	269	5.4	1.7	0	0%	269	3.0	1.3	5	2%	391	10.1	1.4	232	41%					
Rwanda-rukara	270	4.0	1.6	80	30%	270	1.8	1.0	80	30%	365	10.3	1.8	242	34%					
Senegal	392	9.3	4.5	2	1%	392	5.3	3.2	6	2%	392	10.1	2.4	209	47%					
Uganda-Kampala	721	7.6	3.6	8	1%	714	4.3	2.5	26	4%	728	11.6	1.3	646	11%	717	210	86	186	26%
Zanzibar-Kivunge	297	6.2	3.1	18	6%	297	3.5	2.3	22	7%	297	8.8	1.5	66	78%					
Zanzibar-Micheweni	105	6.4	1.5	0	0%	105	3.0	1.2	0	0%	105	8.1	1.5	14	87%					

Total	3830	7.8	3.9	120	3%	3043	4.0	2.6	170	6%	4283	10.3	2.0	2607	39%	1414	195	90	450	32%

### Frequency of events

During follow-up (any time, all observations), abnormal values occurred in 2% (224/10,677) for leucopaenia, 8% (621/8,232) for neutropaenia, 40% (5,102/12,888) for anaemia, 19% (685/3,514) for thrombocytopaenia.

Of all the above events, the proportion of those that were severe (grade 3 and 4) was 8% (19/224) for leucopaenia, 3% (18/621) for neutropaenia, 28% (1,412/5,102) for anaemia, and 15% (100/685) for thrombocytopaenia.

Overall, the proportion of patients with severe leucopaenia was lower on AS&AQ than other treatments (*p *= 0.023 stratified by site, accounted for by a higher frequency with AL in Zanzibar) while no difference was detected for severe neutropaenia, severe anaemia, and severe thrombocytopaenia (*p *= 0.804, *p *= 0.800, *p *= 0.470, respectively, stratified by site).

### Haematological treatment emergent adverse events (TEAEs)

The occurrence of TEAEs for neutropaenia, anaemia and thrombocytopaenia could be calculated for 1192, 2063 and 1375 patients respectively who had both baseline and follow-up values (through Day 28 for neutropaenia and anaemia, and Day 14 for thrombocytopaenia) (Table 5). In those treated with AS&AQ, the overall incidence of TEAEs was 11%, 20% and 6%, respectively.

**Table 5:** Post-treatment haematologic adverse event (AE), treatment emergent AEs (TEAE), between Day 0 and Day 28

Condition	Treatment	Normal on admission		TEAE (n)		AE (n)		TEAE	AE
		n	%	n	N (total)	n	N (total)	%	%
Neutropaenia	AS&AQ	1350	95%	144	1309	169	1323	11%	13%
	Other artemisinin	919	95%	113	944	130	951	12%	14%
	Non-artemisinin	602	92%	63	536	80	538	12%	15%

Anaemia	AS&AQ	1141	59%	343	1750	693	1751	20%	40%
	Other artemisinin	876	59%	328	1462	650	1462	22%	44%
	Non-artemisinin	590	68%	29	717	82	718	4%	11%

Thrombocytopaenia	AS&AQ	343	73%	28	463	39	463	6%	8%
	Other artemisinin	436	64%	44	672	68	672	7%	10%

No difference was observed for neutropaenia between AS&AQ compared to other artemisinin treatments (*p *= 0.475) or non-artemisinin treatments (*p *= 0.642), and for thrombocytopaenia compared to other artemisinin treatments (*p *= 0.734). In contrast with the incidence of TEAE for anaemia that was higher in AS&AQ or other artemisinin groups compared to non-artemisinin treatments (*p *= 0.001, for both comparisons).

### Descriptive paired analysis (baseline *vs *follow-up)

#### Single-agent comparators

##### Amodiaquine (AQ) mono-therapy (Additional file 1)

Two RCTs comparing AS&AQ (n = 464) to AQ alone (n = 457) conducted at five sites [5,17] showed no increased risk when adding AS to AQ. There was:

i. no significant variation in WCC through Day 28 in both groups, and no difference in variations between AS&AQ and AQ (*p *= 0.882). In each group one patient was leucopaenic on admission and recovered; one in the AQ group became leucopaenic and none in the AS&AQ.

ii. a significant decrease in neutrophils by Day 7 and 14 (-17% and -37%, no data on Day 28) for AS&AQ and on Day 7 for AQ alone (-21%), but no difference between the two groups; there was one neutropaenic patient in the AQ group who recovered and none in the AS&AQ group. Only one patient from the AS&AQ group became neutropaenic.

iii. a significant increase in haemoglobin in both groups on Days 14 and 28 (final gain 13% and 10%, respectively) with no significant difference between the two groups. The proportion of patients recovering from anaemia by Day 28 was higher with AQ (100%, 19/19) compared to AS+AQ (80%, 24/30, *p *= 0.037). The proportions of patients becoming anaemic were not different between AQ (6%, 3/49) and AS&AQ (2%, 1/48, *p *= 0.385).

##### Artesunate (AS) mono-therapy (Additional file 1)

One RCT in Bougoula, Mali [13] comparing AS+AQ (n = 252) to AS (n = 252) showed no increased risk (and a lower risk of anaemia) when adding AQ to AS. There was:

i. a significant decrease in WCC in both groups without difference between groups through Day 28; there was no leucopaenic patient in both groups during the follow-up.

ii. an increase in platelets in both groups without difference between the two groups; 92% (11/12) in the AS+AQ, and 75% (3/4) of the patients in the AS group recovered (*p *= 0.802); 15% (4/27) and 40% (2/5), respectively became thrombocytopaenic (*p *= 0.291).

iii. a significant transient decrease in haemoglobin by Day 7 with AS+AQ and AS alike; by Day 28, the gain in haemoglobin was greater in patients treated with AS+AQ (+10%, 95%CI 7%-13%) than AS alone (+7%, 95%CI 3%-7%, *p *= 0.031). The proportion of patients recovering from anaemia was slightly higher in AS+AQ (64%, 62/97) but not significantly different from AS (55%, 51/93, *p *= 0.203). The risk of becoming anaemic was higher with AS alone (15%, 18/120) than AS+AQ (7%, 9/138, OR 2.53, 1.09-5.87, *p *= 0.026).

### ACT comparators

#### Artesunate plus sulphadoxine/pyrimethamine (AS+SP) (Additional file 1)

In Mali [13], AS+AQ (n = 252) was compared to AS+SP (n = 249). No difference in WCC and platelets changes was detected between the two groups during follow-up. There was a significant transient decline in haemoglobin by Day 7, and an increase in both groups through Day 28. The proportions of patients recovering from anaemia by Day 28 in AS&AQ (64%, 62/97) compared to AS+SP (62%, 67/108) and becoming anaemic in AS&AQ (7%, 9/138) compared to AS+SP (9%, 11/128) groups were not different (*p *= 0.781 and *p *= 0.522, respectively, for both comparisons).

#### Artemether-lumefantrine (AL) combination (Additional file 1)

Two RCTs comparing AS&AQ (n = 1070) to AL (n = 762) in seven sites [10,16] showed:

i. no change in WCC through Day 28 with AL and a significant decrease with AS&AQ on Days 14 and 28 which was significantly greater than AL on Day 28 (-7%, 95%CI -4% to -10% *vs *0%, 95%CI -5%-4%, *p *= 0.017); however, Day 28 WCC were not different between the two groups (7.5 × 10^9^/L and 7.4 × 10^9^/L, *p *= 0.402). The proportion of patients recovering from leucopaenia on admission was not different (AS&AQ = 100%, 10/10, AL = 90%, 9/10). In AS&AQ < 1% (2/753), and 1% (5/461) of the patients in AL group became leucopaenic (*p *= 0.092).

ii. a significant decrease in neutrophil counts with both drugs at each time point (Days 7, 14 and 28) which was significantly greater with AS&AQ than AL on Days 14 and 28 (Day 28 variations were -35%, 95%CI -30%-39% with AS&AQ *vs *-28%, 95%CI -22% to -34% with AL, *p *= 0.005); however, Day 28 neutrophil counts were not different (3.1 and 3.0, *p *= 0.861). The proportion of patients recovering from pre-treatment neutropaenia was similar in the two groups (AS&AQ = 89%, 17/19, AL = 85%, 11/13), as well as the proportion of patients becoming neutropaenic (6%, 44/718 and 6%, 27/442, respectively, *p *= 0.989).

iii. increased lymphocyte counts by over 40% in both the AS&AQ and AL group; no difference in variations was detected between the two groups (for all comparisons).

iv. by Day 7, a more marked decrease in haemoglobin in the AS&AQ compared to the AL group (*p *= 0.001), and a smaller increase by Day 14 (*p *= 0.049), but by Day 28 no significant difference in haemoglobin levels and the proportions of patients recovering from anaemia (54%, 190/353 and 56%, 138/246, *p *= 0.582, respectively) or becoming anaemic (10%, 39/409 and 8% 19/225, *p *= 0.648, respectively).

v. a significant increase in platelet counts of about 90% was observed in both groups. All patients recovered in both groups with no patients developing thrombocytopaenia.

#### Dihydroartemisinin-piperaquine (DP) (Additional file 1)

One RCT in Rwanda [15] comparing AS+AQ (n = 252) to DP (n = 252) provided for haematologic data on admission and Day 14.

i. WCC decreased significantly in the DP group but not in the AS+AQ group, but the difference between the two groups was statistically non-significant (*p *= 0.24). The proportion of patients recovering from leucopaenia was higher in the AS+AQ (65%, 22/34) than in the DP group (37%, 14/37, *p *= 0.051). However by Day 14, the prevalence rates of leucopaenic patients were not different between groups (8%, 19/247; 9%, 23/248, respectively, *p *= 0.528).

ii. the prevalence of neutropaenia on admission was 15% (38/252) in the AS+AQ group and 10% (24/251) in the DP group (*p *= 0.060); at Day 14 the prevalence rates were also not different in the AS+AQ group (15%, 37/247) compared to the DP group (10%, 24/248, *p *= 0.073). A significant decrease in neutrophils was observed in both groups. No difference was found between the two groups in the fall in neutrophil counts (*p *= 0.800) or the proportions of patients recovering (*p *= 0.720).

iii. Patients on AS+AQ had a faster haemoglobin recovery (+10%, 95%CI 7%-13%) by Day 14 than those treated with DP (+6%, 95%CI 3%-9%, *p *= 0.040). The proportion of patients recovering from anaemia was not different between the two groups (*p *= 0.45), but the risk of becoming anaemic with DP (9%, 14/164) was higher (but not significantly different) than with AS+AQ (4%, 6/160, OR 2.40, 95%CI 0.90-6.40, *p *= 0.073).

### Non-artemisinin containing comparators

#### Amodiaquine plus sulphadoxine/pyrimethamine (AQ+SP) (Additional file 1)

AS+AQ (n = 494) was compared to AQ+SP (n = 527) in Rwanda and Uganda [14,15] where data were only available on admission and on Day 14. By day 14, WCC slightly decreased with a significant decrease of neutrophils in both groups; haemoglobin and platelets significantly increased in both groups. No difference was detected between treatment groups for all haematologic parameters studied (WCC, neutrophils, platelets).

### Adjusted risks and trends

Large inter-individual differences in haematologic outcomes were detected (*p *= 0.001, LM test) requiring the use of random effects. All the following analysis used multivariate logistic regression with random intercept on individuals including all potential risk factors. During the drug-free, post-treatment follow-up, it was found that:

i. the risk of leucopaenia decreased (AOR 0.96, 95%CI 0.94-0.98, *p *= 0.001), and was lower in older patients (AOR 0.92, 95%CI 0.86-0.98, *p *= 0.013) and higher in patients with higher baseline parasitaemia (AOR 1.80, 95%CI 1.21-2.69, *p *= 0.001). No difference was detected between AS&AQ and comparator treatments (AL, *p *= 0.09; AS, *p *= 0.18; AS+SP, *p *= 0.94; AQ+SP, *p *= 0.63) and treatment outcome (success or parasitological reappearance) (*p *= 0.11). The risk of severe (grade 3 and 4) leucopaenia was lower in older patients (*p *= 0.031) and higher in patients treated from AL (*p *= 0.031, all cases occurring in Zanzibar) compared to AS&AQ.

ii. the risk of neutropaenia increased (AOR 1.03, 95%CI 1.02-1.04, *p *= 0.001); older patients were at lower risks (AOR 0.92, 95%CI 0.90-0.95, *p *= 0.001). No difference was detected between AS&AQ and either AQ (*p *= 0.16), AL (*p *= 0.56), AQ+SP (*p *= 0.98), or DP (*p *= 0.08), or with respect to treatment outcome (*p *= 0.67) or baseline parasitaemia (*p *= 0.56). No risk of severe neutropaenia (grade 3 and 4) was detected.

iii. the risk of anaemia decreased (AOR 0.91, 95%CI 0.90-0.92, *p *= 0.001); patients with higher baseline parasitaemia (AOR 1.53, 95%CI 1.30-1.80, *p *= 0.001) and those experiencing recurrent parasitaemia (AOR 1.98, 95%CI 1.35-2.92, *p *= 0.001) were at higher risk, while older patients were at lower risk (AOR 0.67, 95%CI 0.64-0.70, *p *= 0.001). No difference was detected between AS&AQ and either AL (*p *= 0.67), AS + SP (*p *= 0.21), DP (*p *= 0.69), AQ+SP (*p *= 0.22), AQ (*p *= 0.48), or AS (*p *= 0.08). No risk of severe anaemia (grade 3 and 4) was detected.

iv. the risk of thrombocytopaenia decreased (AOR 0.88, 95%CI 0.87-0.90, *p *= 0.001); older patients (AOR 0.92, 95%CI 0.88-0.96, *p *= 0.001) were at lower risks compared to younger patients; patients with higher baseline parasitaemia (AOR 1.46, 95%CI 1.25-1.70, *p *= 0.001), treated in Uganda with AL (AOR 1.77, 95%CI 1.03-3.04, *p *= 0.040) or AQ+SP (AOR 1.95, 95%CI 1.15-3.32, *p *= 0.013) were at higher risks for thrombocytopaenia by Day 14. In Mali, the risks of patients treated with AS (*p *= 0.64) or AS+SP (*p *= 0.47) were not significantly different from AS&AQ. Patients with a parasitological recurrence were at higher risks for thrombocytopaenia (AOR 3.55, 95%CI 1.29-9.79, *p *= 0.014). There were no data for AQ and DP. Similarly, the risk of severe thrombocytopaenia dropped significantly during follow-up (*p *= 0.025); younger patients (*p *= 0.001) and patients treated with AS (*p *= 0.002) were at significant higher risks compared to AS&AQ; no difference was detected for the other treatments.

## Discussion

Safety in general and laboratory data in particular are under-reported in malaria, and limited information can be derived from aggregated data meta-analyses. This study obviates some of these shortcomings by collecting and analysing individual data on a substantial number of patients (about 4,500) from RCTs of loose or fixed-dose artesunate-amodiaquine combinations (about 2,000) *vs *single-agent and combination (artemisinin- or non artemisinin-containing) therapies. Studies were identified through a systematic review of the literature conducted in 2008 and contacting investigators who may be willing to contribute their data [12]. A few additional comparative trials for the treatment of acute falciparum malaria in Sub-Saharan Africa [11,22,23] could not be included. Ideally, a database to monitor safety should be established and constantly updated.

In contrast with meta-analysis of aggregated study reports, individual patient data permit analysis of haematologic parameters as both continuous and categorical (e.g. using common toxicity grades) variables, as well as multivariate analyses including covariates such as individuals, study site, age, baseline parasitaemia, treatment outcome, and treatment group. As a result, conclusions can be drawn as to the contributions to haematologic changes (both toxicity and recovery) of either components of the combination (artesunate and amodiaquine) both individually and together.

Overall, there appears to be no obvious, specific haematologic risk systematically associated with artesunate-amodiaquine as compared to single-agent and other combination (with or without an artemisinin) therapies.

Knowledge of the haematologic changes occurring during acute malaria and recovery is incomplete, limiting our ability to analyse and understand drug-induced changes. A recent analysis from this group of data in African children under five years of age treated for *P. falciparum *[24] showed that acute malaria (pre-treatment) induced a moderate increase in white cells counts (WCC, +5%) resulting from an increase in neutrophils (+43%) that was proportionally larger than the decrease in lymphocytes counts (-16%); haemoglobin and platelets decreased (-13% and -49%). Post-treatment, although there was a small decrease in WCC, the risks of leucopaenia decreased while the risk of neutropaenia increased.

Differently from the above-cited study, the present one had patients of all ages (although 72% were under 5 years old) and no lymphocyte counts. Six of the seven trials of this analysis are in common with the previous analysis [24]. Burkina Faso [6] was not included here since the trial compared two ASAQ combinations, and a study conducted in Rwanda comparing AS+AQ to AQ was added [17]. However, as the present analysis was not restricted to children under five years of age, only around half (54%, 2447/4502) of the patients were in common in both analyses.

In the present study including also about one-third of adults, acute malaria (pre-treatment) was associated with a very low risk of leucopaenia (3%) and neutropaenia (6%); instead, anaemia (about 60%) and thrombocytopaenia (about one-third) were common. Also, older patients were at lower risk for leucopaenia, neutropaenia, anaemia, and thrombocytopaenia, contrary to findings in the narrower group of children under five years of age, for whom no age-effect was apparent except for anaemia [24].

Post-treatment, the risk of leucopaenia, anaemia and thrombocytopaenia decreased, while the risk of neutropaenia increased over time. When interpreting this finding, it should be noted that: (i) the risk post-treatment includes the prevalence of all the events occurring throughout the 28-day follow-up period and (ii) the time trends (analysed as a continuous variable in days) are minimal in particular for leucopaenia and neutropaenia. The respective adjusted risk (AOR) 95%CIs were 0.94-0.98 and 1.02-1.04.

### WCC and leucopaenia

Leucopaenia was infrequent both at baseline and post-treatment. White blood cells total counts without differential counts appear to be uninformative as they will not capture larger variations in neutrophil counts that are partly compensated by opposite variations in lymphocytes (not assessed here, but seen in under five years old [24]). Baseline WCC counts were overall within the normal ranges but conditions may vary; in one site (Rukara, Rwanda) 30% of patients had leucopaenia pre-treatment [15,17]. Post-treatment, WCC values decreased minimally or remained constant and within normal ranges resulting in a low frequency of leucopaenia (2%) occurring during follow-up, without significant differences between treatments. Older patients were at lower risk, while higher baseline parasitaemia increased the risk. WCC was not a good surrogate for neutropaenia.

### Neutrophil counts and neutropaenia

Baseline neutrophil counts were overall normal (6% neutropaenia, consistent with 7% found in under five years old [24]), but with wide variations across sites (30% in Rukara, Rwanda [15]). There were fewer patients with differential counts recorded post-treatment than patients with total WCC (73% and 86% on Days 14, and 28, respectively). Neutropaenia post-treatment was more frequent than leucopaenia. No significant difference in neutrophil counts was apparent between treatments except for a greater drop (approximately by one-third) by Day 14 with AS&AQ than with artemether-lumefantrine on Day 14, but Day 28 counts were not different.

Age appeared to protect against neutropaenia (here like in [24]), but here there was no association between neutropaenia and baseline parasitaemia or type of treatment (while in under five years old the risk of neutropaenia was lower in case of higher baseline parasitaemia and ACT treatment).

### Haemoglobin and anaemia

Anaemia was frequent before treatment (around 60%, on average, and up to 90% in Cameroon and Uganda-Kampala) [14,16] and decreased significantly over time after treatment in all groups. The net gain in haemoglobin by Day 28 was consistently around 10% over the baseline value with most of the treatments. Only artesunate mono-therapy [13] and dihydroartemisinin-piperaquine by Day 14 [15] had a significantly lower gain than with AS&AQ. With all treatments, patients with high parasitaemia and parasitological reappearance during the follow-up were at higher risk of anaemia, while older age protected against anaemia irrespective of the parasitaemia (only the latter found in under five years old [24]).

Artemisinin compounds have been shown to induce reticulocytopaenia both in experimental and clinical conditions potentially by suppressing erythroblasts and that malaria itself could protect against reticulocytopaenia [25]. None of these studies reported reticulocyte counts, and only three studies had a comparator without artesunate (two *vs *amodiaquine alone and one *vs *amodiaquine plus SP). No clear indication results from these studies. In two studies [5,17] the proportion of patients recovering from anaemia by Day 28 was higher after treatment with amodiaquine alone than when combined with artesunate while the proportions of patients becoming anaemic was not different; conversely, no differences were seen between treatments combining amodiaquine with artesunate or SP.

### Platelets and thrombocytopaenia

Only two studies recorded platelets in Mali [13] and in Uganda [14], so it is difficult to generalize. The prevalence of thrombocytopaenia decreased post-treatment (from 32% pre-treatment to 9% on Day 14). A reduction of the risk was observed up to Day 14 corresponding with the upward trend in platelets counts observed in [24]. Baseline parasitaemia did not affect platelets variations, but parasitological reappearance did, and there was a treatment effect in both datasets. A smaller increase in platelets corresponding to a greater risk of thrombocytopaenia was observed in Uganda with AQ+SP (*p *= 0.040) and AL (*p *= 0.013) compared to the AS&AQ, while no difference between groups was detected in Mali.

### Adverse events and treatment-related adverse events

Deviation of laboratory values from normality is graded according to widely accepted severity criteria [23]; however, establishing causality (drug-event relationship) is often a challenge for physicians. The incidence of TEAEs was calculated in the subgroup of patients with values on both Day 0 and post-treatment follow-up through Day 28 (only Day 14 was available for platelets) and found no difference between treatments for neutropaenia, and thrombocytopaenic but a higher risk of TEAEs for anaemia with ACT than with non-artemisinin combinations. TEAEs express more reliably the deterioration of conditions as they exclude AEs, which occurred already at the same intensity before treatment. This analysis was also conservative, as all events, occurring at any time post-treatment, were counted, irrespective of whether the parameter improved later on.

The size of this database (relatively large), the spectrum representation (locations and ages) and the analyses done (multivariate) are all positive features and advantages over single-site papers or meta-analyses of aggregate data. However, a note of caution is needed as to how to interpret these results. The absence of a signal does not mean that there is no risk, or certainty about no excess risk (over other treatments). Rare events will require a very large sample size that was not available here. The sample size is further reduced for individual variables (in particular white blood cells differential counts, hence neutropaenia); also, all variables were not assessed uniformly at the same time. There is also the issue of special risk groups, such as HIV-coinfected subjects (high risk for neutropaenia [7,8]) and pregnant women (potential risk for reticulocytopaenia - reviewed in [25]), which are not represented in the population under study here.

## Abbreviations

ACT: Artemisinin Combination Therapy; AE: adverse event; AL: Artemether-Lumefantrine; AQ: amodiaquine; AS: artesunate; DP: Dihydroartemisinin-piperaquine; IPD: Individual Patient Data; LM: Lagrange multiplier test; Max: maximum; Min: minimum; RCT: Randomized Controlled Trial; SD: standard deviation; SP: Sulphadoxine-Pyrimethamine; TEAE: treatment emergent adverse event; WHO: World Health Organization.

## Competing interests

The authors declare that they have no competing interests.

## Authors' contributions

JZ and PO designed the analysis, interpreted the data and prepared the manuscript. All authors read and approved the final manuscript.

## Disclaimer

P. Olliaro is a staff member of the WHO; the authors alone are responsible for the views expressed in this publication and they do not necessarily represent the decisions, policy or views of the WHO.

## Supplementary Material

Additional file 1 **Haematologic paired analysis, RCTs, AS&AQ *vs *comparator groups**. Click here for file
