# Supplementary material for: Comparing changes in haematologic parameters occurring in patients included in randomized controlled trials of artesunate-amodiaquine vs single and combination treatments of uncomplicated falciparum in sub-Saharan Africa
Source: Malar J. 2012 Jan 25;11:25. doi: 10.1186/1475-2875-11-25 (PMC3298482; doi:10.1186/1475-2875-11-25)
Supplement: Additional file 1 — Haematologic paired analysis, RCTs, AS&AQ vs comparator groups. [file 1475-2875-11-25-S1.DOC]

# Additional file 1: Haematologic paired analysis, RCTs, AS&AQ vs. comparators

#

Table 1: Haematologic paired analysis, RCTs, AS&AQ vs. AQ

| **Parameter** | |  | Day 0 - Day 7 | | Day 0 - Day 14 | | Day 0 - Day 28 | |
| --- | --- | --- | --- | --- | --- | --- | --- | --- |
|  |  |  | AS&AQ | AQ | AS&AQ | AQ | AS&AQ | AQ |
| **WCC (x10^9/L)** | N paired | | 45 | 39 | 23 | 14 | 75 | 71 |
|  | Admission | mean | 9.0 | 7.8 | 8.1 | 7.1 | 7.9 | 7.6 |
|  |  | sd | 3.6 | 3.2 | 3.3 | 2.3 | 3.0 | 2.8 |
|  | Follow-up | mean | 9.7 | 8.0 | 7.8 | 7.5 | 7.8 | 7.5 |
|  |  | sd | 4.1 | 3.2 | 3.3 | 2.7 | 2.6 | 2.2 |
|  | Relative difference | % | 9% | 3% | -3% | 6% | -0.3% | -2% |
|  |  | lower 95%CI | -4% | -11% | -22% | -18% | -10% | -12% |
|  |  | upper 95%CI | 22% | 18% | 15% | 29% | 9% | 8% |
|  | P value (paired) | | 0.240 | 0.638 | 0.768 | 0.634 | 0.946 | 0.690 |
|  | P value (difference) | | | 0.854 |  | 0.632 |  | 0.882 |
| **Neutrophils (x10^9/L)** | N paired | | 43 | 37 | 22 | 14 |  |  |
|  | Admission | mean | 5.0 | 4.5 | 4.1 | 3.6 |  |  |
|  |  | sd | 2.4 | 3.1 | 2.0 | 2.4 |  |  |
|  | Follow-up | mean | 4.2 | 3.5 | 2.6 | 3.3 |  |  |
|  |  | sd | 1.8 | 3.1 | 1.5 | 2.4 |  |  |
|  | Relative difference | % | -17% | -21% | -37% | -9% |  |  |
|  |  | lower 95%CI | -34% | -47% | -64% | -54% |  |  |
|  |  | upper 95%CI | 1% | 4% | -10% | 36% |  |  |
|  | P value (paired) | | 0.022 | 0.027 | 0.023 | 0.690 |  |  |
|  | P value (difference) | | | 0.668 |  | 0.102 |  |  |
| **Haemoglobin (g/dL)** | N paired | | 60 | 63 | 157 | 147 | 78 | 68 |
|  | Admission | mean | 9.6 | 9.7 | 10.1 | 10.3 | 10.4 | 10.7 |
|  |  | sd | 1.9 | 2.0 | 1.7 | 1.7 | 1.9 | 1.8 |
|  | Follow-up | mean | 9.7 | 9.9 | 10.6 | 11.0 | 11.7 | 11.7 |
|  |  | sd | 1.6 | 2.0 | 1.2 | 1.2 | 1.2 | 1.1 |
|  | Relative difference | % | 1% | 2% | 6% | 6% | 13% | 10% |
|  |  | lower 95%CI | -6% | -5% | 2% | 3% | 8% | 5% |
|  |  | upper 95%CI | 8% | 9% | 9% | 10% | 19% | 15% |
|  | P value (paired) | | 0.684 | 0.436 | 0.000 | 0.000 | 0.000 | 0.000 |
|  | P value (difference) | | | 0.980 |  | 0.909 |  | 0.175 |

Legend. AS: artesunate and amodiaquine; AQ: amodiaquine; CI: confidence interval;

Table 2: Haematologic paired analysis, RCTs, AS&AQ vs. AS+SP vs. AS

|  |  |  | Day 0 - Day 7 | | | Day 0 - Day 14 | | | Day 0 - Day 28 | | |
| --- | --- | --- | --- | --- | --- | --- | --- | --- | --- | --- | --- |
| **Parameter** |  |  | AS&AQ | AS+SP | AS | AS&AQ | AS+SP | AS | AS&AQ | AS+SP | AS |
| **WCC (x10^9/L)** | N paired | | 250 | 246 | 251 | 229 | 225 | 233 | 14 | 7 | 7 |
|  | Admission | mean | 10.5 | 10.2 | 10.5 | 10.5 | 10.2 | 10.6 | 10.4 | 9.8 | 10.3 |
|  |  | sd | 4.2 | 4.1 | 4.8 | 4.2 | 4.0 | 4.8 | 4.1 | 3.7 | 3.0 |
|  | Follow-up | mean | 9.8 | 9.8 | 9.6 | 8.3 | 8.3 | 8.5 | 8.3 | 9.6 | 7.8 |
|  |  | sd | 3.6 | 9.1 | 3.8 | 3.1 | 3.1 | 2.9 | 1.6 | 1.7 | 2.2 |
|  | Relative difference % | % | -6% | -11% | -8% | -21% | -19% | -20% | -20% | -2% | -24% |
|  |  | lower 95%CI | -11% | -16% | -13% | -26% | -16% | -15% | -37% | -27% | -47% |
|  |  | upper 95%CI | -2% | -6% | -4% | -23% | -14% | -25% | -3% | 23% | 0% |
|  | P-value |  | 0.024 | 0.000 | 0.004 | 0.000 | 0.000 | 0.000 | 0.091 | 0.910 | 0.022 |
|  | Difference (AS&AQ ref.) | | | 0.155 | 0.356 |  | 0.506 | 0.615 |  | 0.255 | 0.488 |
| **Haemoglobin (x10^9/L)** | N paired | | 249 | 247 | 251 | 247 | 245 | 233 | 235 | 236 | 213 |
|  | Admission | mean | 10.3 | 10.2 | 10.2 | 10.3 | 10.2 | 10.2 | 10.2 | 10.2 | 10.2 |
|  |  | sd | 1.8 | 1.9 | 1.8 | 1.8 | 1.9 | 1.8 | 1.8 | 1.9 | 1.8 |
|  | Follow-up | mean | 9.8 | 9.8 | 9.6 | 10.6 | 10.4 | 10.3 | 11.3 | 11.1 | 10.9 |
|  |  | sd | 1.7 | 9.7 | 1.5 | 1.5 | 1.7 | 1.4 | 1.6 | 1.6 | 1.6 |
|  | Relative difference % | % | -4% | -5% | -6% | 3% | 2% | 1% | 10% | 8% | 7% |
|  |  | lower 95%CI | -7% | -8% | -3% | 0% | 6% | 4% | 7% | 5% | 3% |
|  |  | upper 95%CI | -1% | -2% | -9% | -2% | 5% | -3% | 13% | 12% | 10% |
|  | P-value |  | 0.000 | 0.000 | 0.004 | 0.000 | 0.039 | 0.000 | 0.000 | 0.000 | 0.022 |
|  | Difference (AS&AQ ref.) | | | 0.493 | 0.170 |  | 0.429 | 0.054 |  | 0.284 | 0.031 |
| **Platelets (x10^9/L)** | N paired | | 231 | 227 | 233 | 229 | 225 | 233 | 19 | 11 | 9 |
|  | Admission | mean | 178 | 179 | 183 | 179 | 179 | 183 | 161 | 203 | 172 |
|  |  | sd | 88 | 86 | 105 | 88 | 86 | 105 | 88 | 92 | 96 |
|  | Follow-up | mean | 292 | 292 | 272 | 269 | 270 | 272 | 281 | 265 | 226 |
|  |  | sd | 132 | 305 | 121 | 133 | 124 | 121 | 88 | 99 | 121 |
|  | Relative difference % | % | 64% | 71% | 49% | 51% | 51% | 49% | 74% | 31% | 32% |
|  |  | lower 95%CI | 62% | 69% | 50% | 49% | 52% | 50% | 69% | 25% | 24% |
|  |  | upper 95%CI | 66% | 72% | 47% | 49% | 52% | 47% | 80% | 36% | 40% |
|  | P-value |  | 0.000 | 0.000 | 0.000 | 0.000 | 0.000 | 0.000 | 0.001 | 0.223 | 0.283 |
|  | Difference (AS&AQ ref.) | | | 0.575 | 0.681 |  | 0.800 | 0.918 |  | 0.350 | 0.263 |

Table 3: Day 28 haematologic paired analysis, RCTs, AS&AQ vs. AL (WCC, neutrophils, lymphocytes)

| **Parameter** | | | Day 0 - Day 7 | | Day 0 - Day 14 | | Day 0 - Day 28 | |
| --- | --- | --- | --- | --- | --- | --- | --- | --- |
|  |  | | AS&AQ | AL | AS&AQ | AL | AS&AQ | AL |
| **WCC (x10^9/L)** | N paired | | 766 | 482 | 430 | 402 | 763 | 471 |
|  | Admission | mean | 8.0 | 7.5 | 7.2 | 6.8 | 8.1 | 7.4 |
|  |  | sd | 3.6 | 3.1 | 3.3 | 3.7 | 3.7 | 3.4 |
|  | Follow-up | mean | 8.0 | 8.0 | 6.9 | 6.9 | 7.5 | 7.4 |
|  |  | sd | 3.3 | 7.6 | 2.3 | 2.6 | 2.9 | 3.3 |
|  | Relative difference | % | 0% | 0% | -4% | 3% | -7% | 0% |
|  |  | lower 95%CI | -3% | -4% | -9% | 0% | -10% | -5% |
|  |  | upper 95%CI | 3% | 5% | -3% | 8% | -4% | 4% |
|  | P-value | | 0.948 | 0.841 | 0.039 | 0.379 | 0.000 | 0.845 |
|  | Difference AS&AQ vs AL | | | 0.510 |  | 0.898 |  | 0.017 |
| **Neutrophils (x10^9/L)** | N paired | | 735 | 465 | 427 | 399 | 737 | 455 |
|  | Admission | mean | 4.6 | 4.3 | 4.0 | 3.6 | 4.7 | 4.2 |
|  |  | sd | 2.7 | 1.5 | 2.6 | 2.1 | 2.8 | 2.6 |
|  | Follow-up | mean | 3.3 | 3.3 | 2.6 | 2.7 | 3.1 | 3.0 |
|  |  | sd | 1.5 | 3.0 | 1.2 | 1.2 | 1.7 | 1.5 |
|  | Relative difference | % | -28% | -30% | -36% | -26% | -35% | -28% |
|  |  | lower 95%CI | -33% | -35% | -43% | -30% | -39% | -34% |
|  |  | upper 95%CI | -24% | -25% | -33% | -19% | -30% | -22% |
|  | P-value | | 0.000 | 0.000 | 0.000 | 0.000 | 0.000 | 0.000 |
|  | Difference AS&AQ vs AL | | | 0.942 |  | 0.009 |  | 0.005 |
| **Lymphocytes (x10^9/L)** | N paired | | 563 | 285 | 109 | 61 | 560 | 276 |
|  | Admission | mean | 2.7 | 2.7 | 1.9 | 2.1 | 2.7 | 2.7 |
|  |  | sd | 1.8 | 3.6 | 1.1 | 1.3 | 1.9 | 1.7 |
|  | Follow-up | mean | 3.9 | 3.9 | 3.3 | 3.5 | 3.8 | 3.8 |
|  |  | sd | 2.1 | 4.1 | 1.3 | 1.4 | 1.9 | 2.6 |
|  | Relative difference | % | 45% | 53% | 79% | 65% | 41% | 42% |
|  |  | lower 95%CI | 36% | 37% | 56% | 101% | 32% | 29% |
|  |  | upper 95%CI | 54% | 69% | 37% | 93% | 49% | 55% |
|  | P-value | | 0.000 | 0.000 | 0.000 | 0.000 | 0.000 | 0.000 |
|  | Difference AS&AQ vs AL | | | 0.426 |  | 0.776 |  | 0.504 |

Table 4: Day 28 haematologic paired analysis, RCTs, AS&AQ vs. AL (haemoglobin, platelet)

| **Parameter** |  |  | Day 0 - Day 7 | | Day 0 - Day 14 | | Day 0 - Day 28 | |
| --- | --- | --- | --- | --- | --- | --- | --- | --- |
|  |  | | AS&AQ | AL | AS&AQ | AL | AS&AQ | AL |
| **Haemoglobin (g/dL)** | N paired | | 766 | 482 | 436 | 409 | 762 | 471 |
|  | Admission | mean | 10.1 | 9.9 | 10.1 | 10.1 | 10.1 | 10.0 |
|  |  | sd | 2.3 | 1.9 | 2.1 | 2.0 | 2.3 | 2.3 |
|  | Follow-up | mean | 9.6 | 9.6 | 10.4 | 10.6 | 11.0 | 10.9 |
|  |  | sd | 2.0 | 9.7 | 1.6 | 1.5 | 1.8 | 1.6 |
|  | Relative difference | % | -5% | -2% | 3% | 4% | 9% | 10% |
|  |  | lower 95%CI | -7% | -4% | 0% | 5% | 7% | 7% |
|  |  | upper 95%CI | -3% | 1% | 2% | 7% | 11% | 7% |
|  | P-value |  | 0.000 | 0.004 | 0.000 | 0.000 | 0.000 | 0.000 |
|  | Difference AS&AQ vs AL | | | 0.001 |  | 0.049 |  | 0.531 |
| **Platelets (x10^9/L)** | N paired | |  |  | 230 | 208 |  |  |
|  | Admission | mean |  |  | 226 | 201 |  |  |
|  |  | sd |  |  | 91 | 79 |  |  |
|  | Follow-up | mean |  |  | 417 | 399 |  |  |
|  |  | sd |  |  | 117 | 120 |  |  |
|  | Relative difference | % |  |  | 84% | 99% |  |  |
|  |  | lower 95%CI | |  | 83% | 86% |  |  |
|  |  | upper 95%CI | |  | 97% | 100% |  |  |
|  | P-value |  |  |  | 0.000 | 0.000 |  |  |
|  | Difference AS&AQ vs AL | | |  |  | 0.609 |  |  |

Table 5: Day 14 haematologic paired analysis. AS&AQ vs. DP, Rwanda

| **Parameter** |  | Day 0 - Day 14 | | | | | |
| --- | --- | --- | --- | --- | --- | --- | --- |
|  |  | **WCC  (x10^9/L)** | | **Neutrophils (x10^9/L)** | | **Haemoglobin (g/dL)** | |
|  | | AS+AQ | DP | AS+AQ | DP | AS+AQ | DP |
| N paired | | 247 | 248 | 247 | 248 | 247 | 247 |
| Admission | mean | 5.3 | 5.3 | 2.8 | 2.8 | 10.3 | 10.5 |
|  | sd | 2.3 | 2.1 | 1.7 | 1.5 | 1.8 | 1.6 |
| Follow-up | mean | 5.1 | 5.0 | 2.0 | 2.0 | 11.3 | 11.1 |
|  | sd | 2.0 | 1.9 | 1.1 | 1.0 | 1.2 | 1.2 |
| Relative difference | % | -2% | -5% | -29% | -27% | 10% | 6% |
|  | lower 95%CI | -9% | -11% | -39% | -37% | 7% | 3% |
|  | upper 95%CI | 5% | 0% | -18% | -17% | 13% | 9% |
| P value (paired) | | 0.358 | 0.024 | 0.000 | 0.000 | 0.000 | 0.000 |
| P value (difference) | | | 0.264 |  | 0.800 |  | 0.040 |

Table 6: Day 14 haematologic paired analysis, RCTs, AS&AQ vs. AQ+SP

|  | | Day 0 - Day 14 | | | | | | | |
| --- | --- | --- | --- | --- | --- | --- | --- | --- | --- |
|  |  | **WCC  (x10^9/L)** | | **Neutrophils (x10^9/L)** | | **Haemoglobin (g/dL)** | | **Platelets  (x10^9/L)** | |
|  | | AS+AQ | AQ+SP | AS+AQ | AQ+SP | AS+AQ | AQ+SP | AS+AQ | AQ+SP |
| N paired | | 476 | 499 | 474 | 499 | 482 | 506 | 230 | 250 |
| Admission | mean | 6.5 | 6.3 | 3.6 | 3.5 | 10.9 | 11.1 | 226 | 202 |
|  | sd | 3.3 | 2.9 | 2.5 | 2.2 | 1.7 | 1.7 | 91 | 80 |
| Follow-up | mean | 6.3 | 6.2 | 2.3 | 2.2 | 11.4 | 11.7 | 417 | 398 |
|  | sd | 2.5 | 2.7 | 1.2 | 1.1 | 1.1 | 1.2 | 117 | 126 |
| Relative difference | % | -3% | -2% | -37% | -37% | 5% | 5% | 84% | 97% |
|  | lower 95%CI | -8% | -6% | -44% | -44% | 3% | 3% | 83% | 96% |
|  | upper 95%CI | 2% | 3% | -30% | -31% | 7% | 7% | 86% | 98% |
| P value (paired) | | 0.131 | 0.354 | 0.000 | 0.000 | 0.000 | 0.000 | 0.000 | 0.000 |
| P value (difference) | | | 0.898 |  | 0.940 |  | 0.181 |  | 0.616 |

Legend. Only Day 14 data available for paired analysis. AS: artesunate; AQ: amodiaquine; SP: sulphadoxine/pyrimethamine; CI: confidence interval;
